# Inflammatory macrophage phenotype in BTBR T+tf/J mice

**DOI:** 10.3389/fnins.2013.00158

**Published:** 2013-09-17

**Authors:** Charity E. Onore, Milo Careaga, Brooke A. Babineau, Jared J. Schwartzer, Robert F. Berman, Paul Ashwood

**Affiliations:** ^1^The MIND Institute, University of CaliforniaDavis, CA, USA; ^2^Department of Medical Microbiology and Immunology, University of CaliforniaDavis, CA, USA; ^3^Department of Pediatrics, University of CaliforniaSan Francisco, CA, USA; ^4^Institute for Human Genetics, University of CaliforniaSan Francisco, CA, USA; ^5^Department of Neurological Surgery, University of CaliforniaDavis, CA, USA

**Keywords:** autism, BTBR, behavior, immune system, inflammation, macrophage, M1, M2

## Abstract

Although autism is a behaviorally defined disorder, many studies report an association with increased pro-inflammatory cytokine production. Recent characterization of the BTBR T+tf/J (BTBR) inbred mouse strain has revealed several behavioral characteristics including social deficits, repetitive behavior, and atypical vocalizations which may be relevant to autism. We therefore hypothesized that, asocial BTBR mice, which exhibit autism-like behaviors, may have an inflammatory immune profile similar to that observed in children with autism. The objectives of this study were to characterize the myeloid immune profile of BTBR mice and to explore their associations with autism-relevant behaviors. C57BL/6J (C57) mice and BTBR mice were tested for social interest and repetitive self-grooming behavior. Cytokine production was measured in bone-marrow derived macrophages incubated for 24 h in either growth media alone, LPS, IL-4/LPS, or IFNγ/LPS to ascertain any M1/M2 skewing. After LPS stimulation, BTBR macrophages produced higher levels of IL-6, MCP-1, and MIP-1α and lower IL-10 (*p* < 0.01) than C57 mice, suggesting an exaggerated inflammatory profile. After exposure to IL-4/LPS BTBR macrophages produced less IL-10 (*p* < 0.01) than C57 macrophages and more IL-12p40 (*p* < 0.01) suggesting poor M2 polarization. Levels of IL-12(p70) (*p* < 0.05) were higher in BTBR macrophages after IFNγ/LPS stimulation, suggesting enhanced M1 polarization. We further observed a positive correlation between grooming frequency, and production of IL-12(p40), IL-12p70, IL-6, and TNFα (*p* < 0.05) after treatment with IFNγ/LPS across both strains. Collectively, these data suggest that the asocial BTBR mouse strain exhibits a more inflammatory, or M1, macrophage profile in comparison to the social C57 strain. We have further demonstrated a relationship between this relative increase in inflammation and repetitive grooming behavior, which may have relevance to repetitive and stereotyped behavior of autism.

## Background

Autism is a behaviorally defined disorder without a known physiological cause. This disorder is characterized by social deficits, communication deficits, and the presence of repetitive or stereotyped behaviors (APA, 2000). Several studies have attempted to identify a genetic cause for autism (Yrigollen et al., 2008; Voineagu et al., 2011; Lintas et al., 2012); however, the majority of autism cases remain idiopathic in nature (Abrahams and Geschwind, 2008). Although the genetic cause for autism remains undetermined, there is increasing evidence that immune function may play a role in the disorder. A number of studies have identified trends in abnormal immune function in individuals with autism including a trend toward high cytokine production and atypical immune cell function (Ashwood et al., 2006, 2011a,b,c; Enstrom et al., 2010). There is further evidence suggesting that many of these atypical immune profiles are associated with worsening autism-associated behaviors (Onore et al., 2012).

Immune abnormalities in autism spectrum disorders have been characterized in a number of studies, including evidence of increased microglia and astroglia activation in the brain, as well as increased levels of interferon (IFN)γ, interleukin (IL)-1β, IL-6, IL-12, tumor necrosis factor (TNF)-α, and macrophage chemotactic protein (MCP)-1 in brain tissue and cerebral spinal fluid (Vargas et al., 2005; Li et al., 2009; Morgan et al., 2010). In parallel with findings within the central nervous system (CNS), increased plasma levels of these cytokines including IL-1β, IL-6, IL-12p40, and MCP-1 have been reported as well (Ashwood et al., 2011b). Of note, peripheral plasma levels of these cytokines were associated with worsening autism-associated behaviors in the areas of communication and social interaction, suggesting a relationship between peripheral immune activity and behavioral symptoms of the disorder (Ashwood et al., 2011b,c).

Given the unknown pathophysiology of autism and highly variable genetics of the disorder, an animal model that exhibits behavioral characteristics with relevance to autism-features rather than a direct genetic link may be useful for examining potential relationship between physiology and behavior. The BTBR T+tf/J mouse (referred to as BTBR here-in) is an inbred strain that has recently been described to exhibit behaviors relevant to autism, including asocial behavior, repetitive behavior, and atypical vocalizations (Moy et al., 2007; McFarlane et al., 2008; Scattoni et al., 2008, 2011). There is also evidence to suggest that the BTBR mouse exhibits increased ERK signaling (Zou et al., 2011), a common finding in individuals with Fragile X Syndrome, one of the leading known genetic risk factors for autism. However, little is currently known about the relationship between BTBR associated behavior and immunity in the BTBR mouse. Recent research has outlined differences in immune function including increased titers of immunoglobulin (Ig) and inflammatory cytokine levels in brain tissue, and greater susceptibility to listeriosis than C57Bl/6J mice (referred to as C57 here-in) (Heo et al., 2011). Immunity against listeriosis is largely based on the ability of macrophages to polarize to an inflammatory (M1) profile (Pfeffer et al., 1993; Jouanguy et al., 1999; Benoit et al., 2008). This data suggests immunological differences between the two strains and may implicate aberrant macrophage function, but there is as yet no direct evidence of a relationship between the function of the immune cells and behavioral symptoms in the BTBR mouse.

We hypothesized that there are associations between myeloid inflammation and autism relevant behaviors in the BTBR mouse. To further elucidate the relationship between inflammation and behavior, and to examine the role of M1/M2 polarization in BTBR, we analyzed individual mice for social interaction and repetitive grooming behavior and measured macrophage cytokine production *in vitro.* Bone marrow-derived macrophages were generated for each mouse and tested for inflammatory and anti-inflammatory cytokine responses. In addition, we further characterized immune function by testing the ability of BTBR macrophages to polarize to pro-inflammatory IL-12 high (M1) and anti-inflammatory IL-10 high (M2) macrophage subtypes *in vitro* (Mantovani et al., 2005).

## Methods

### Mice

C57Bl/6J (C57) mice (Jackson Laboratory-West, Sacramento, CA), BTBR T^+^tf/J (BTBR) mice (Jackson Laboratory, Bar Harbor, ME), and 129/SvImJ (129) mice (Jackson Laboratory) were maintained by the Campus Laboratory Animal Services, at University of California, Davis at ambient room temperature on a 12 h light/dark cycle. Food and water were provided *ad libitum*. All procedures were performed with approval by the University of California, Davis Institutional Animal Care and Use Committee and in accordance with the guidelines provided by the National Institutes of Health for the humane treatment of animals. Social approach and self-grooming assays were conducted in dedicated behavioral testing rooms during the standard light phase, usually between 1000 and 1500 h. Mice of the 129 strain were used as novel mouse controls in the social approach testing paradigm. Mice were deeply anesthetized with isofluorane and euthanized by decapitation. Femurs and tibia were aseptically removed and stored in RPMI 1640 (Life Technologies) media supplemented with 10% FBS, 100 IU/ml penicillin, 100 IU/ml streptomycin, 25 μg/ml gentimycin (Sigma) prior to processing. For this study, nine C57 mice (male, age 10–12 weeks) and seven BTBR mice (male, age 10–12 weeks), were utilized.

### Social approach

Social approach was assayed in an automated three-chambered apparatus (NIMH Research Services Branch, Bethesda, MD) using methods previously described (Crawley, 2007; Yang et al., 2007, 2009, 2011a,b; Chadman et al., 2008; McFarlane et al., 2008; Moy et al., 2008; Silverman et al., 2010, 2011). Briefly, the apparatus was a rectangular, three-chambered box made of clear polycarbonate. Photocells embedded in the doorways automatically detected entries between chambers and the amount of time spent in each chamber by the subject mouse. The test session began with a 10 min habituation session in the center chamber only, followed by a 10 min habituation to all three empty chambers. At the completion of the second habituation phase, the subject mouse was returned to the center chamber and a clean novel object (wire cup) was placed in one of the side chambers and a novel 129 mouse of similar age and weight was placed in an identical wire cup located in the other side chamber. After both stimuli were positioned, the subject mouse was allowed access to all three chambers for 10 min. Trials were video recorded and time spent sniffing the novel object and time spent sniffing the novel mouse were later scored by a trained observer using a hand-held stopwatch. Performance on social approach was plotted as a “social score,” which is equal to the amount of time (in seconds) the experimental animal spent sniffing the novel mouse minus the time spent sniffing the novel object. Therefore, high positive scores indicate high sociability, and low or negative scores indicate low sociability.

### Spontaneous self grooming

Mice were scored for spontaneous self-grooming behaviors as described previously (Yang et al., 2007, 2009; McFarlane et al., 2008; Silverman et al., 2010). Briefly, each mouse was given a 10 min habituation in a clean, empty mouse cage and then video recorded for 10 min. The video recorded session was scored for cumulative time spent grooming all body regions by a trained observer using a stopwatch. Differences in color and markings between the inbred strains prevented fully blind ratings. However, the distinguishing features of the strain were less visible in the video recordings, which is why this method was chosen over live scoring. The animals' behavior in the spontaneous self-grooming tasks was plotted as “grooming” which indicates the amount of time (in seconds) the experimental animal spent grooming itself during the 10-min observation phase.

### Generation of macrophage media

Confluent L929 Cells (ATCC, Manassas, VA) were cultured for 7 days in complete Dubelcco's modified Eagles media (DMEM) F-12 (Life Technologies, Carlsbad, CA) supplemented with 10% low endotoxin, heat inactivated fetal bovine serum (FBS) (Life technologies), 100 IU/ml penicillin, and 100 IU/ml streptomycin (Sigma, St Louis, MO). The resulting L929 conditioned media was passed through a 2 μm filter (Millipore, Billerica, MA) to remove cellular debris and ensure sterility. To create macrophage media, complete DMEM was supplemented 10% with filtered L929 conditioned media. L929 conditioned media was stored at −20°C for less than 60 days before single thaw and use.

### Bone marrow-derived macrophage generation

Legs with fur and skin removed, including femur and tibia from each mouse, were washed twice in sterile cold (4°C) Hanks buffered saline solution (HBSS) (Mediatech, Herndon, VA). Tissue was removed from the bones with sterile scissors and forceps, and bones were washed in 10 mls cold HBSS. The proximal and distal ends of both the femur and tibia were removed, and the lumens of the femurs and tibia were flushed with 10 mls of cold HBSS using a 25 gauge needle (BD Medical, Franklin Lakes, NJ). Dislodged bone marrow was agitated by aspiration and ejected with a 22 gauge needle (BD Medical). The resulting cell suspension was filtered through a 100 μm nylon mesh (BD Biosciences, Carlsbad, CA). Cells were pelleted by centrifugation at 500 g for 5 min and resuspended in macrophage media to a concentration of 1 × 10^5^ cells/ml, plated in sterile non-cell culture treated petri dishes (BD Biosciences) at a volume of 10 mls per dish and incubated for 3 days at 37°C, 5% CO_2_. Five mls of macrophage media was then added to each dish and cells were incubated for a further 4 days at 37°C, 5% CO_2_ for a total incubation time of 7 days. Petri dishes containing adherent, mature bone marrow-derived macrophages were washed with 10 mls cold HBSS, and incubated with 3 mls Cell Stripper™ buffer (Mediatech) per plate, for 5 min at 37°C, 5% CO_2_. Following incubation, cells were dislodged from the petri dishes by gentle pipetting, diluted in an equal amount of complete DMEM, and pelleted by centrifugation at 400 g for 5 min. Cells were resuspended at 1 × 10^6^ cell/ml in complete DMEM, and 1 ml was plated in 12-well sterile tissue culture plate (Greiner Bio-One, Monroe, NC) and allowed to adhere overnight at 37°C, 5% CO_2_. Following adhesion of bone marrow-derived macrophages, complete DMEM was aspirated and replaced with 1 ml/well of 1 × 10^6^ cells with the following eight conditions: media alone, 2 ng/ml recombinant mouse IL-4 (R&D, Minneapolis, MN), 150 ng/ml recombinant mouse IFNγ (R&D), 10 ng/ml lipopolysaccharide (LPS) (Sigma), 2 ng/ml recombinant mouse IL-4 with 10 ng/ml LPS, or 150 ng/ml recombinant mouse IFNγ with 10 ng/ml LPS. Cells were incubated under these conditions for 24 h, at which point supernatants were collected and stored at −80°C until assayed.

### Cytokine measurement

The quantification of the cytokines Granulocyte Macrophage-Colony Stimulating Factor (GM-CSF), IFNγ, IL-1β, IL-6, IL-10, IL-13, IFNγ induced protein (IP)-10 (CXCL10), MCP-1 (CCL2), TNFα, macrophage inflammatory protein (MIP)-1α (CCL3), and MIP-1β (CCL4) in supernatants was determined using mouse specific Milliplex™ multiplexing bead immunoassays (Millipore, Billerica, MA). The cytokines IL-12p40 and IL-12p70 were measured using Bioplex™ multiplexing bead immunoassays (Bio-Rad Laboratories, Hercules, CA). Samples were run in accordance with the instructions of the manufactures protocol. In brief, 25 μL of supernatant was incubated with antibody-coupled beads. After a series of washes, a biotinylated detection antibody was added to the beads, and the reaction mixture was detected by the addition of streptavidin conjugated to phycoerythrin. The bead sets were analyzed using a flow-based Luminex™ 100 suspension array system (Bio-Plex 200; Bio-Rad Laboratories). Unknown sample cytokine concentrations were calculated by Bio-Plex Manager software using a standard curve derived from the known reference cytokine concentrations supplied by the manufacturer. A five-parameter model was used to calculate final concentrations and values are expressed in pg/ml. The sensitivity of this multiplex immunoassay allowed the detection of cytokine concentrations with the following minimal detectable limits: IL-1β (2 pg/ml), IL-6 (1.8 pg/ml), IL-10 (3.3 pg/ml), IL-12(p40) (1.53 pg/ml), IL-12(p70) (1.62 pg/ml), IFNγ (0.9 pg/ml), IP-10 (0.6 pg/ml), MCP-1 (5.3 pg/ml), MIP-1α (8.7 pg/ml), MIP-1β (10.1 pg/ml), and TNFα (1 pg/ml).

### Statistical analysis

Statistical analysis to compare cytokine levels between BTBR and C57 groups was conducted with Mann–Whitney test. Correlation analysis was performed with Spearman correlation analysis. All analyses were two-tailed, and values of *p* < 0.05 were considered statistically significant. Unadjusted *p*-values are presented for multiplex cytokine data (Rothman, 1990). Medians and interquartile ranges (IQR) are reported for all measured cytokines and chemokines. All analyses were conducted with GraphPad Prism statistical software (GraphPad Software Inc., San Diego, CA).

## Results

### Cytokine responses

To determine the cytokine response to LPS (a gram negative bacterial endotoxin) with and without polarization, bone marrow macrophages were incubated in media alone, IL-4 (a M2 polarizing cytokine), or IFNγ (a M1 polarizing cytokine) with LPS. Even without presence of polarizing cytokine or LPS stimulus, BTBR macrophages exhibit a trend toward higher inflammatory cytokine production, with significantly higher production of IL-12(p40) [C57 median ± IQR: 15.30 ± 7.56; median ± IQR: 20.58 ± 7.56; *p* = 0.0485] (Table 1). Following stimulation with LPS, BTBR macrophages produce significantly higher levels of inflammatory cytokines IL-6 [C57 median ± IQR: 1254 ± 760.2; BTBR median ± IQR: 3112 ± 1189; *p* = 0.0003], IL-12(p40) [C57 median ± IQR: 446.4 ± 322.5; BTBR median ± IQR: 2779 ± 1652; *p* = 0.0002], MCP-1 [C57 median ± IQR: 750.4 ± 442.6; BTBR median ± IQR: 3117 ± 1036; *p* = 0.0002], IP-10 [C57 median ± IQR: 8875 ± 2859; BTBR median ± IQR: 14535 ± 1294; *p* = 0.0007], and MIP-1α [C57 median ± IQR: 1945 ± 713; BTBR median ± IQR: 4342 ± 2828; *p* = 0.0002] and lower levels of anti-inflammatory cytokine IL-10 [C57 median ± IQR: 448.4 ± 307.6; BTBR median ± IQR: 284.2 ± 195.2; *p* = 0.0229] (Table 1).

**Table 1:** **Macrophage cytokine levels in unstimulated and LPS stimulated culture conditions**.

**Cytokines pg/ml**	**Unstimulated: Median (IQR)**			**LPS: Median (IQR)**		
	**C57**	**BTBR**	***p*-value**	**C57**	**BTBR**	***p*-value**
IL-1β	3.9 (14.39)	BLD	N/A	BLD	16.56 (38.26)	0.351
IL-6	0.23 (2.22)	BLD	N/A	1254 (760.2)	3112 (1189)	0.0003^*^
IL-10	BLD	BLD	N/A	448.4 (307.6)	284.2 (195.2)	0.023^*^
IL-12p40	15.3 (7.56)	20.58 (7.56)	0.049^*^	446.4 (322.5)	2779 (1652)	0.0002^*^
IL-12p70	BLD	BLD	N/A	BLD	56.81 (56.81)	N/A
IP-10	65.45 (30.94)	70.17 (93.81)	0.142	8875 (2859)	14535 (1294)	0.0007^*^
MCP-1	BLD	15.63 (32.74)	N/A	750.4 (442.6)	3117 (1036)	0.0002^*^
TNF-α	BLD	0.13 (5.1)	N/A	448.3 (407.5)	595.2 (217.2)	0.408
MIP-1α	39.27 (22.41)	BLD	N/A	1945 (713)	4342 (2828)	0.0002^*^
MIP-1β	63.35 (36.58)	56.76 (83.06)	0.671	10214 (2530)	23060 (9262)	0.0003^*^

Median cytokine measurements and interquartile ranges (IQR) in pg/ml. All p-values were calculated by two-tailed Mann Whitney U-test.

* Significant p-values; BLD: Below level of detection; N/A: Not applicable, p-values could not be calculated.

**Figure 1:**
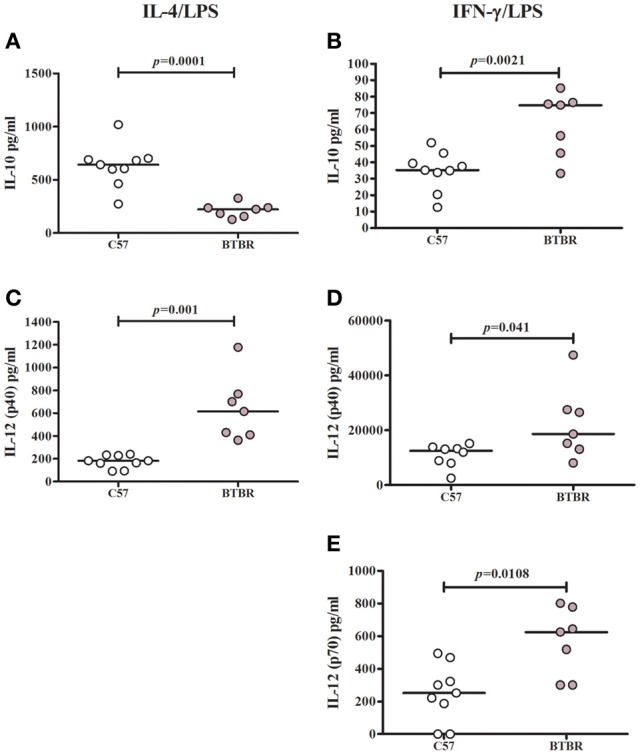
**M1 and M2 cytokine polarization in BTBR and C57 macrophages**. Scatter graphs and median values of IL-10 **(A,B)**, IL-12p40 **(C,D)** and IL-12p70 **(E)** after IL-4/LPS incubation **(A,C)** and IFNγ/LPS incubation **(B,D,E)**. IL-12(p70) values were below observable levels in IL-4/LPS stimulated samples. *p*-values were calculated with Mann–Whitney test.

To determine whether BTBR macrophages are more inclined toward a pro-inflammatory M1 phenotype, we measured the levels of M1 and M2 associated cytokines following polarization with IL-4 and LPS to induce a M2 phenotype or IFNγ and LPS to induce a M1 phenotype. Our initial experiments revealed treatment with IL-4 alone was not sufficient to induce a cytokine response distinguishable from that of media alone. Following stimulation with IL-4 and LPS, BTBR macrophages produce significantly lower levels of IL-10 [C57 median ± IQR: 641.9 ± 164.2; BTBR median ± IQR: 222.6 ± 83.4; *p* = 0. 0.0003], a M2 cytokine, and higher IL-12p40 [C57 median ± IQR: 182.3 ± 102.6; BTBR median ± IQR: 613.8 ± 359.4; *p* = 0.001], IL-6 [C57 median ± IQR: 558.6 ± 288.1; BTBR median ± IQR: 1264 ± 330; *p* = 0.0052], and MIP-1α [C57 median ± IQR: 2551 ± 1150; BTBR median ± IQR: 4179 ± 4467; *p* = 0.0021]. Following stimulation with IFNγ and LPS, BTBR macrophages produce significantly higher levels of IL-12(p40) [C57 median ± IQR: 12496 ± 5494; BTBR median ± IQR: 18607 ± 326.6; *p* = 0.0401], IL-12(p70) [C57 median ± IQR: 251.9 ± 302.17; BTBR median ± IQR: 625.5 ± 477.1; *p* = 0.0108], and IP-10 [C57 median ± IQR: 3464 ± 1953; BTBR median ± IQR: 7497 ± 3553; *p* = 0.0002] but also more IL-10 [C57 median ± IQR: 35.24 ± 15.36; BTBR median ± IQR: 74.8 ± 30.9; *p* = 0.0115] and MCP-1 [C57 median ± IQR: 1238 ± 802; BTBR median ± IQR: 1954 ± 752; *p* = 0.0012] (Table 2). BTBR macrophages appeared to exhibit an overall trend toward increased M1 polarization (Figure 1).

**Table 2:** **Macrophage cytokine levels in IL-4/LPS and IFNγ/LPS stimulated culture conditions**.

**Cytokines pg/ml**	**IL-4/LPS: Median (IQR)**			**IFNγ/LPS: Median (IQR)**		
	**C57**	**BTBR**	***p*-value**	**C57**	**BTBR**	***p*-value**
IL-1β	20.04 (32.39)	BLD	0.252	BLD	46.36 (28.18)	N/A
IL-6	558.6 (288.1)	1264 (330)	0.005^*^	10680 (8041)	10667 (5695)	0.919
IL-10	641.9 (164.2)	222.6 (83.4)	0.0003^*^	35.24 (15.36)	74.8 (30.9)	0.012^*^
IL-12p40	182.3 (102.6)	613.8 (359.4)	0.001^*^	12496 (5494)	18607(326.6)	0.040^*^
IL-12p70	BLD	BLD	N/A	251.9 (302.17)	625.5 (477.1)	0.011^*^
IP-10	3464 (1953)	7497 (3553)	0.0002^*^	18319 (7141)	16726 (10137)	0.351
MCP-1	2692 (682)	3373 (1065)	0.351	1238 (802)	1954 (752)	0.001^*^
TNF-α	418.2 (292.6)	498.5 (282)	0.351	975.2 (451.8)	1393 (974)	0.071
MIP-1α	2551 (1150)	4179 (4467)	0.002^*^	872.3 (321.6)	1134 (330.2)	0.091
MIP-1β	4264 (2097)	6136 (17675)	0.055	4019 (1492)	5791 (3024)	0.071

Median cytokine measurements and interquartile ranges (IQR) in pg/ml. All p-values were calculated by two-tailed Mann Whitney U-test.

* Significant p-values; BLD: Below level of detection; N/A: Not applicable, p-values could not be calculated.

### Behavioral and immune correlations

**Figure 2:**
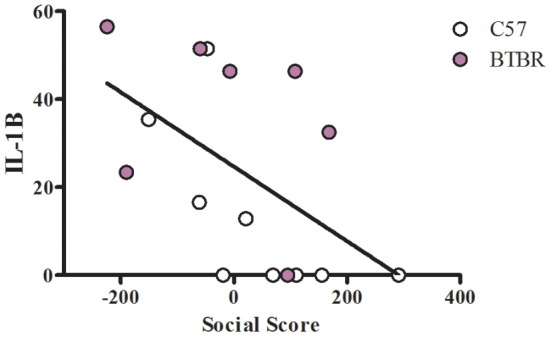
**Social correlation in IFNγ/LPS stimulated macrophages**. Scatter graphs and linear regression of IL-1β in IFNγ stimulated macrophages. Correlation coefficients (*r*-values) and *p*-values were calculated with Spearman's correlation analysis. C57 *r*-value: −0.80, *p*-value: 0.009^*^; BTBR *r*-value: −0.43, *p*-value: 0.333; Across strains *r*-value: −0.56, *p*-value: 0.023^*^; ^*^significant *p*-values.

**Figure 3:**
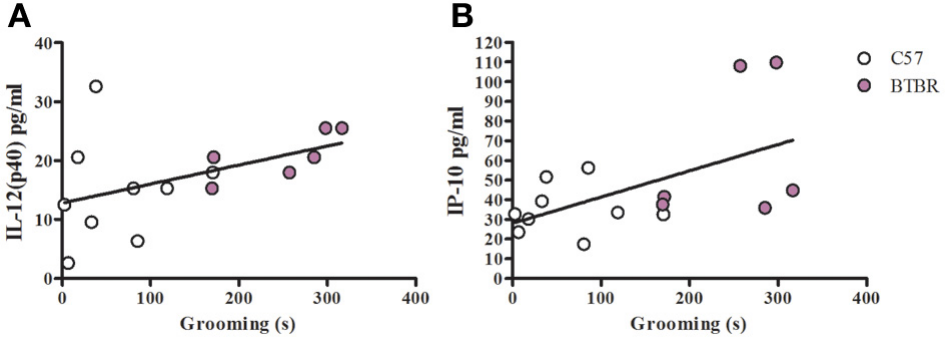
**Grooming correlation in unstimulated macrophages**. Scatter graphs and linear regression of IL-12(p40) **(A)** and IP-10 **(B)** in unstimulated macrophages. Correlation coefficients (*r*-value) and *p*-values were calculated with Spearman's correlation analysis. **(A)** C57 *r*-value: 0.27, *p*-value: 0.493; BTBR *r*-value: 0.88, *p*-value: 0.033^*^; Across strains *r*-value; 0.58, *p*-value: 0.024^*^ **(B)** C57 *r*-value: 0.23, *p*-value: 0.546; BTBR *r*-value: 0.43, *p*-value: 0.397; Across strains *r*-value: 0.55, *p*-value: 0.032^*^; ^*^significant *p*-values.

**Figure 4:**
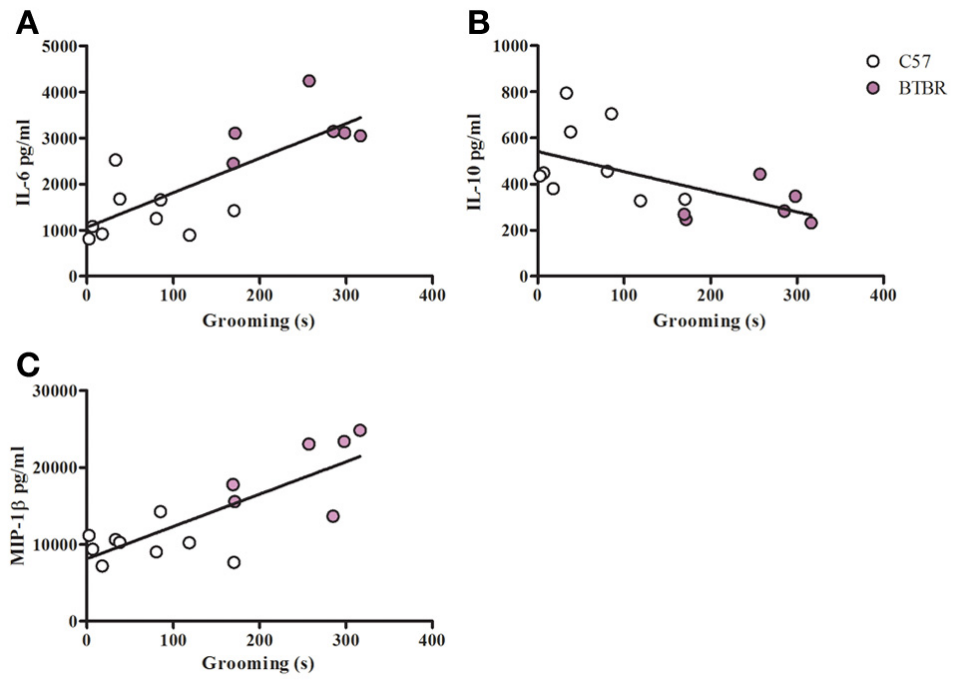
**Grooming correlation in LPS stimulated macrophages**. Scatter graphs and linear regression of IL-6 **(A)**, IL-10 **(B)**, and **(C)** MIP-1β in LPS stimulated macrophages. Correlation coefficients (*r*-values) and *p*-values were calculated with Spearman's correlation analysis. **(A)** C57 *r*-value: 0.30, *p*-value: 0.433; BTBR *r*-value: 0.20, *p*-value: 0.704; Across strains *r*-value: 0.77, *p*-value: 0.001^*^ **(B)** C57 *r*-value: −0.22, *p*-value: 0.576; BTBR *r*-value: −0.09, *p*-value: 0.872; Across strains *r*-value: −0.63, *p*-value: 0.012^*^ **(C)** C57 *r*-value: −0.15, *p*-value: 0.708; BTBR *r*-value: 0.60, *p*-value: 0.241; Across strains *r*- value: 0.67, *p*-value: 0.006; ^*^significant *p*-values.

**Figure 5:**
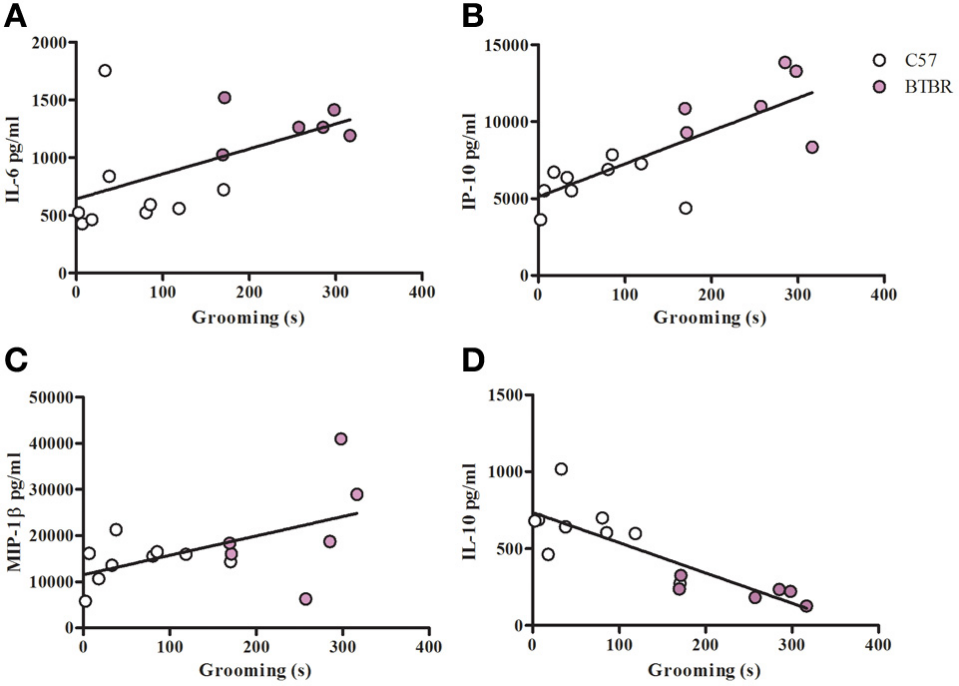
**Grooming correlation in IL-4/LPS stimulated macrophages**. Scatter graphs and linear regression of IL-6 **(A)**, IP-10 **(B)**, MIP-1β **(C)**, and IL-10 **(D)** in IL-4/LPS stimulated macrophages. Correlation coefficients (*r*-value) and *p*-values were calculated with Spearman's correlation analysis. **(A)** C57 *r*-value: 0.43, *p*-value: 0.244; BTBR *r*-value: 0.09, *p*-value: 0.872; Across strains *r*-value: 0.63, *p*-value: 0.012^*^ **(B)** C57 *r*-value: 0.40, *p*-value: 0.291; BTBR *r*-value: 0.02, *p*-value: 1; Across strains *r*-value: 0.78, *p*-value: 0.0007^*^ **(C)** C57 *r*-value: 0.38, *p*-value: 0.312; BTBR *r*-value: 0.71, *p*-value: 0.136; Across strains *r*-value: 0.53, *p*-value: 0.042^*^ **(D)** C57 *r*-value: −0.48, *p*-value: 0.188; BTBR *r*-value: −0.77, *p*-value: 0.072; Across strains *r*-value: −0.86, *p*-value: 0.0001^*^; ^*^significant *p*-values.

**Figure 6:**
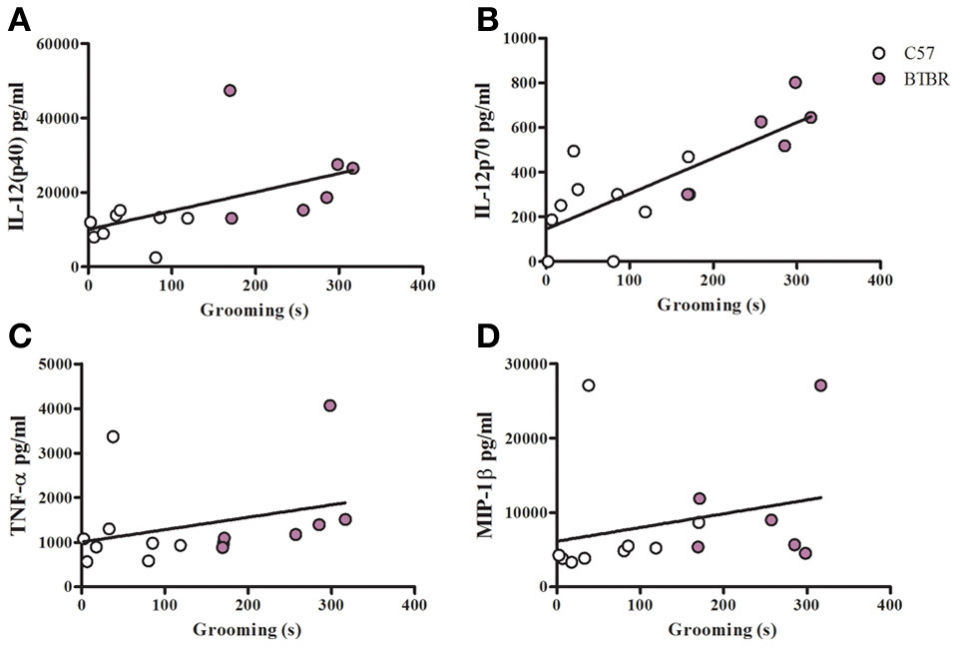
**Grooming correlation in IFNγ/LPS stimulated macrophages**. Scatter graphs and linear regression of IL-12(p40) **(A)**, IL-12(p70) **(B)**, TNFα **(C)**, and MIP-1β **(D)** in IFNγ/LPS stimulated macrophages. Correlation coefficients (*r*-values) and *p*-values were calculated with Spearman's correlation analysis. **(A)** C57 *r*-value: 0.26, *p*-value: 0.536; BTBR *r*-value: 0.09, *p*-value: 0.919; Across strains *r*-value: 0.72, *p*-value: 0.004^*^ **(B)** C57 *r*-value: 0.39, *p*-value: 0.291; BTBR *r*-value: 0.87, *p*-value: 0.033^*^; Across strains *r*-value: 0.77, *p*-value: 0.001^*^ **(C)** C57 *r*-value: 0.02, *p*-value: 0.966; BTBR *r*-value: 0.94, *p*-value: 0.005^*^; Across strains *r*-value 0.51, *p*-value: 0.005^*^ **(D)** C57 *r*-value: 0.70, *p*-value: 0.036^*^; BTBR *r*-value: 0.20, *p*-value: 0.704; Across strains *r*- value: 0.63, *p*-value: 0.011^*^; ^*^significant *p*-values.

To examine a potential relationship between the immune profile observed in BTBR macrophages, and the impaired social interaction and repetitive grooming associated with the BTBR strain, we tested for associations by Spearman analysis. There was very little association between cytokine profiles and social behavior in the BTBR mouse; however, for macrophage cell cultures, IL-1β demonstrated a negative association with sociability after IFNγ/LPS incubation [*r* = −0.56; *p* = 0.023] such that as IL-1β levels increased social approach decreased (Figure 2). In contrast, grooming behavior was associated with a large number of cytokines including associations with IL-12(p40) [*r* = 0.58; *p* = 0.033] and IP-10 [*r* = 0.55; *p* = 0.032] in unstimulated macrophages (Figure 3) suggesting that as cytokine levels increased there was an increase in repetitive behaviors. Following stimulation with LPS, inflammatory cytokines IL-6 [*r* = 0.77; *p* = 0.001] and MIP-1β [*r* = 0.67; *p* = 0.006] were positively associated with grooming behavior, while the anti-inflammatory M2 associated cytokine IL-10 demonstrated a negative association with grooming [*r* = −0.63; *p* = 0.012] (Figure 4). This data suggests that inflammatory cytokines are associated with more impaired repetitive behavior whereas anti-inflammatory cytokines are associated with improvement in behaviors. In polarization conditions such as IL-4/LPS incubation, IL-10 again was negatively associated with grooming [*r* = −0.86; *p* = 0.0001], while IL-6 [*r* = −0.63; *p* = 0.012], IP-10 [*r* = 0.775, *p* = 0.0007], and MIP-1β [*r* = 0.5286, *p* = 0.0428] were positively associated with grooming time (Figure 5). Macrophage cytokines produced following incubation with the M1 polarizing IFNγ/LPS condition including IL-12(p40) [*r* = 0.72; *p* = 0.004], IL-12(p70) [*r* = 0.77; *p* = 0.001], and TNF-α [*r* = 0.63; *p* = 0.011] also positively associated with increased grooming behavior following incubation with the M1 polarizing IFNγ/LPS condition, such that M1 macrophage cytokine production was associated with more impaired behavior. Interestingly, within the BTBR strain alone, IL-12(p70) [*r* = 0.87; *p* = 0.03] and TNF-α [*r* = 0.94; *p* = 0.005] levels were also positively correlated with grooming (Figure 6).

## Discussion

In this study we describe several immune features in the asocial BTBR mouse that differ from social C57 mice including higher production of inflammatory cytokines and chemokines in bone-marrow derived macrophages. BTBR macrophages produced increased levels of IL-6 after LPS stimulation. Even without stimulation, BTBR macrophages produced higher levels of IL-12(p40) and IP-10, suggesting a possible M1 polarization even without the presence of M1 polarizing exposure to IFNγ or LPS. After stimulation with LPS, IL-12(p40) remained higher in BTBR mice, while IL-10 was lower, again suggesting M1 polarization even in the absence of IFNγ. Levels of IL-12(p70) were undetectable in C57 mice, but were detectable in the majority of BTBR samples, suggesting IL-12(p70) levels may also be higher in BTBR mice. However, given that IL-12(p70) were below levels of detection in the C57, statistical analysis was limited and *p*-values could not be calculated. In addition to evidence of M1 polarization, levels of chemokines MIP-1α and MCP-1 were also higher in BTBR macrophages after LPS stimulation suggesting chemotactic responses may also be enhanced in this asocial mouse strain.

Macrophage polarization has many similarities to T helper (T*_H_*) cell subtypes and can be divided into two major groups, denoted M1 and M2 (Mantovani et al., 2005; Mosser and Edwards, 2008). M1 macrophages are IL-12^high^, IL-10^low^, they are higher inflammatory cytokine producers than their M2 counterparts, and promote a T_H_1 response in part through production of IL-12(p70). IL-12(p40) is a subunit of both IL-12(p70) and IL-23, which are both produced in significant quantities by M1 macrophages (Oppmann et al., 2000). While M2 cells can also produce inflammatory cytokines, they tend to produce far less than M1 macrophages, and have an IL-12^low^, IL-10^high^ phenotype, and promote T_H_2 responses. M1 polarization has been implicated in a number of neurological disease including multiple sclerosis and Alzheimer's disease (Mikita et al., 2011), and recent literature suggests that promoting an M2 phenotype may be beneficial to cognitive function (Derecki et al., 2010a). Transplant of wild-type IL-4 competent T cells in to IL-4^−/−^ mice results in improved cognition (Derecki et al., 2010b), and transplant of M2 polarized macrophages results in improved cognitive performance in immune deficient mice (Derecki et al., 2011). Together, this data suggests that M1 macrophage polarization may have detrimental effects on normal brain function whilst M2 can ameliorate some of these effects.

To further examine the proclivity of BTBR macrophages to polarize to an M1 phenotype, C57 and BTBR macrophages were stimulated with LPS in the presence of IFNγ (M1) or IL-4 (M2) (Zhang et al., 2008). Under M1 polarizing conditions, BTBR macrophages produced significantly higher IL-12(p40) and IL-12(p70), mirroring the enhanced M1 polarization. In addition under M2 polarizing conditions, BTBR macrophages produced significantly less IL-10, and significantly more IL-12(p40) suggesting poor relative M2 polarization compared to C57 mice (Figure 1). Collectively, these data suggest that BTBR macrophages are innately M1 skewed, and more inclined to produce M1 associated cytokines such as IL-12(p70).

In this study, we have compared two inbred strains, which were selected for their possession of autism-relevant features or lack thereof (BTBR and C57, respectively) (McFarlane et al., 2008). Given the potential genetic differences between these two inbred strains, it would be possible that these observed immunological differences are circumstantial and unrelated to behavior. To better understand the relationship between autism-like behavior and immune phenotype, we measured social and repetitive behavior in each mouse, and analyzed the correlation between these behavioral measurements and cytokine profiles. We observed only one cytokine correlation with social behavior (Figure 2), which may suggest that social behavior is not heavily affected by immune function within BTBR mice as a group.

Although we did not see strong correlations between social behavior and immune measures in these experiments, the inflammation in BTBR mice may have an impact on fetal development *in-utero*. Maternal immune activation (MIA) in C57 mice results in elevated expression of IL-6, IL-12(p40), IL-12(p70), IL-13, IL-15, IFNγ, TNFα, and IL-10 in pregnant dams (Arrode-Bruses and Bruses, 2012), and leads to deficits in sociability in their offspring (Malkova et al., 2012). The data here-in suggest that BTBR mice innately produce higher levels of many of these cytokines, which may suggest that the maternal environment in which BTBR embryos are exposed to naturally resembles that of the MIA C57 model in terms of increased inflammatory cytokine exposure *in-utero*. Potentially, social impairment is more closely associated with prenatal exposures, while repetitive grooming seems to be related to ongoing elevated inflammation. Additional research, such as prenatal cross-fostering, may help differentiate the roles of the maternal BTBR environment and adult immune profiles in the development of autism-like symptoms in the BTBR mice.

Unlike the social behavior, repetitive grooming behavior correlated with a number of cytokine profiles under all tested culture conditions. IL-12(p40) correlated with increasing grooming behavior of BTBR mice in unstimulated macrophages, and this correlation was also observed across the two strains (Figure 3). Contrary to the positive correlation between IL-12(p40) and repetitive behavior, macrophage derived IL-10 was negatively associated with repetitive grooming across strains in both LPS stimulated and IL-4/LPS stimulated conditions (Figures 4, 5), suggesting a relationship between M1/M2 polarization and repetitive behavior such that M1 was associated with more impairment and M2 with less grooming behaviors. Moreover, both IL-12(p40) and IL-12(p70) showed positive correlations with repetitive grooming post IFNγ/LPS exposure, further supporting a relationship between M1 macrophage cytokines and repetitive grooming.

Of note, a difference in IL-12(p40) was observed between BTBR and C57 strains. We initially measured IL-12(p40) using an allele specific capture antibody, which recognizes several common strains such as C57, BALB/c, and CH3 mice, but does not recognize IL-12(p40) in autoimmune prone strains such as NOD or SJL strains of mice (Ymer et al., 2002). IL-12(p40) from BTBR was also undetectable with this antibody, but was detectable with a non-allele specific anti-IL-12(p40) capture antibody (data not shown). Although we did not test for IL-12(p40) allelic differences between these two strains, an allelic difference may indicate a functional difference for IL-12(p40) *in-vivo.* Interestingly, IL-10 production is also higher in BTBR mice. The synergistic effect of functionally abnormal IL-12p40 allele and high levels of IL-10 production in response to IFNγ may help explain how BTBR mice can both express higher IL-12(p40) and IL-12(p70) than their C57 counterparts, but also be more susceptible to Listeriosis (Heo et al., 2011).

The relationship between repetitive grooming and immune function, particularly in myeloid cells, has been recently illustrated by research in the Hoxb8 deficient mice. Hoxb8 is a myeloid expressed gene associated with repetitive pathological grooming in mice deficient for the gene and excessive grooming is reduced with wild-type bone marrow transplant (Chen et al., 2010). Given this data suggesting that peripherally derived immune cells can have a profound effect on normal grooming behavior, it is possible that ongoing myeloid inflammation may be related to repetitive grooming behavior in animal models. Further evidence that peripherally derived hematopoietic cells can improve behavior was illustrated in a recent paper demonstrating that bone marrow transplant arrested disease development in MeCP2^+/−^ mouse models of Rett's syndrome (Derecki et al., 2012).

Research in children with autism also implicates the role of myeloid cells in the pathology of the disorder. Immunohistochemistry of postmortem brain samples have revealed elevated numbers of microglia in brain parenchyma, and increased perivascular macrophages as well as elevated microglial and perivascular macrophage activation in the brains of children with autism, and increased levels of MCP-1 (Vargas et al., 2005; Morgan et al., 2010). Consistent with findings in the brain, studies examining peripheral myeloid function have revealed increased numbers of circulatory monocytes in the blood and increased cytokine production following TLR4 stimulation in monocytes of children with autism including increased levels of IL-1β, IL-6, and IL-23 and associations with behavioral assessment scores (Sweeten et al., 2003; Jyonouchi et al., 2008; Enstrom et al., 2010). Similar to findings in microglia and monocytes, there is also evidence of atypical distribution of dendritic cell populations in children with autism (Breece et al., 2013), suggesting that many branches of the myeloid system are affected in the disorder. The data described here-in draws many parallels between previously recorded immune phenomena in humans, and the immune profile of the BTBR mouse and warrants a closer examination of neuro-immune interactions in the development of autism spectrum disorders.

In this study, we have demonstrated an inflammatory immune profile is displayed in the asocial BTBR mouse strain as well as associations between inflammation and M1 associated cytokines with repetitive grooming behavior. The immune phenotype of BTBR mice draws several parallels to observation in children with autism particularly high IL-12, IL-6, and MCP-1 production observed in both children with autism and BTBR mice. Although there are caveats in comparing the immunophenotype of two different strains based on behavior, the observed correlations between autism-relevant behaviors and immunological measures suggest the inflammatory phenotype of the BTBR mouse is more than circumstantial. Together these data suggests that the BTBR mouse may possess more than a behavioral similarity to autism in humans, but may also share some physiological symptoms associated with the disorder as well. Although it may not be possible to find a true animal model for autism, the BTBR mouse may prove to be a useful model to study the relationship between inflammation and behavior, the fruits of which may one day be applied to autism and other inflammatory disorders that manifest in behavioral deficits.

### Conflict of interest statement

The authors declare that the research was conducted in the absence of any commercial or financial relationships that could be construed as a potential conflict of interest.
